# Sustained Viral Response and Hematological Adverse Events in Children With Chronic Hepatitis C

**DOI:** 10.5812/hepatmon.851

**Published:** 2012-03-28

**Authors:** Alessandra Vigano, Valeria Manfredini, Gian Vincenzo Zuccotti

**Affiliations:** 1 Department of Pediatrics, Luigi Sacco Hospital, University of Milan, Milan, Italy

**Keywords:** Hepatitis C, Chronic, Child, Interfron-alpha

Dear Editor,

Over the past decades, data from randomized controlled trials have confirmed the use of pegylated interferon alpha (peg-IFNα) plus ribavirin (RBV), as the mainstay of HCV therapy in adults [1]. In children aged 3 years and older, the Food and Drug administration in the United States did not approve the use of this combination therapy until December 2008, and for children in Europe, approval from the European Medicines Agency came a year later in September 2009. Current guidelines for adults suggest starting patients with clinically significant hepatic fibrosis on this treatment, due to the high risk of cirrhosis. Conversely, in pediatric patients, the progression of the condition is slower and the outcomes of the HCV infection are better, so the timing of when to start treatment remains controversial, and no guidelines are currently available for this age group. In addition, as children with chronic HCV infection (CHC) are usually asymptomatic and rarely develop severe liver damage, the possibility of eliciting adverse effects from the current therapies must be balanced appropriately against the benefits. On the other hand, eradicating HCV in order to avert potential hepatic complications in the future, including hepatocarcinoma in later life, is considered by some authors to be a justifiable reason to pursue antiviral therapy in younger individuals as well [2].

The retrospective cohort study recently published in Hepatitis Monthly by Pawlowska et al, analyzed 119 and 51 children with CHC who were treated with; non-pegylated IFNα plus RBV (group 1) or peg-IFNα plus RBV (group 2), respectively. Sustained viral response (SVR), was defined as an undetectable level of HCV-RNA 24 weeks after the end of treatment, and this was achieved by 51% and 47% of patients from group 1 and group 2, respectively. In both groups, the hemoglobin levels as well as the leukocyte and platelet counts, both during treatment and following 12 weeks of therapy, were lower in patients reaching SVR [3]. The rate of SVR reported by the literature in pediatric population for PEG-IFNα plus ribavirin therapy is set between 30 to 100% [1], which is comparable to the rate observed in adults [2]. Among all the factors associated with a SVR, hemoglobin levels, along with leukocyte and platelet counts have previously been described in adult populations [4]. However, in children, the interdependence between the SVR and such hematological characteristics has not been examined in depth. In a previous study [5], Pawlowska et al, had observed a similar earlier response to therapy and ALT normalization in children treated with non-pegylated IFN-alpha and ribavirin who had a decreased leukocyte-count. The recently published study by the same authors has, in our opinion, two strong points. First, it confirms and supports the comparable efficacy of the association of peg-IFNα plus RBV, versus IFNα plus RBV in the pediatric population, data that is consistent with those revised in the most recent adult guidelines. Secondly, it enhances the predictive role for those major hematological disorders, which are commonly considered to be only side effects.
